# Pain Prevention Using Head and Neck Cancer as a Model

**Published:** 2015-01-01

**Authors:** Erin M. McMenamin, Marcia Grant

**Affiliations:** Hospital of the University of Pennsylvania, Philadelphia, Pennsylvania, and City of Hope National Medical Center, Duarte, California

## Abstract

Pain is a common and often debilitating consequence of cancer and its treatment. Efforts to improve pain management for patients diagnosed with cancer have not resulted in widespread patient reports of acceptable management of pain. Patients and providers alike remain opiophobic due to a number of issues, resulting in suboptimal management of pain. Recent literature has revealed that it may be possible to prevent pain related to cancer and its treatment and therefore avoid or decrease the amount of opioids used to treat pain. This may result in better quality of life for patients. Several newer antiepileptic drugs (AEDs) have been found to decrease the perception of pain in a number of patient populations, including those with head and neck cancer. The side-effect profile for the newer AEDs is mild and well tolerated. Future efforts should focus on the use of newer AEDs to prevent pain in other cancer populations, with a focus on ideal dose and scheduling. Once established, recommendations regarding the prevention of pain in patients with cancer can be incorporated into national guidelines.

In 2014, it is estimated that more than 1.6 million people in the United States received new cancer diagnoses and 580,000 cancer-related deaths likely occurred (American Cancer Society [ACS], 2014). Of these patients, more than 60,000 were diagnosed with head and neck cancer (ACS, 2014). In the past, head and neck cancer (HNC) was most often associated with heavy smoking and/or consuming large amounts of alcohol on a regular basis. During the past decade, the demographic for oropharyngeal cancer began changing to younger (average age in the 50s), white males, many of whom have no history of smoking or drinking. This has in large part been due to the development of human papillomavirus (HPV)-related cancer of the tonsils and/or base of the tongue, now responsible for approximately 70% of oropharyngeal head and neck cancer (D’Souza et al., 2007; Kreimer, Clifford, Boyle, & Franceschi, 2005; Chaturvedi et al., 2011).

Treatment for oropharyngeal head and neck cancer has changed as therapies have improved. In the past, patients generally underwent extensive and physically deforming surgery. Many of these patients required postoperative chemotherapy and/or radiation therapy. These treatments often resulted in short- and long-term side effects that impact the quality of life (QOL) of the patient (Richmon, Quon, & Gourin, 2014). More recently, many patients receive treatment that includes definitive CT and RT or, transoral robotic surgery (TORS) to remove the tumor, as well as a selected neck dissection to evaluate the presence of cancer in the regional lymph nodes (Weinstein, O’Malley, Desai, & Quon, 2009). Some surgical candidates also require postoperative chemotherapy and radiation therapy due to adverse pathologic features of the cancer discovered at the time of surgery.

Pain is one of the most prevalent and feared consequences of cancer and its treatment. Regardless of changes and improvements in therapy, patients undergoing treatment for cancer do experience pain. Tumors may invade or compress blood vessels and/or nerves. Treatments are not specific to cancer cells; damage to normal cells results, causing side effects (Epstein et al., 2010). Increased pain in the patient with head and neck cancer is likely to lead to increased utilization of opioid medications, feeding tube insertion, and at times, hospitalization (Murphy et al., 2009). In addition, uncontrolled symptoms lead to treatment breaks, often resulting in decreased disease-free survival and long-term control of the cancer (Withers, Taylor, & Maciejewski, 1988).

Aside from patient-related barriers and despite efforts to educate providers, insufficient progress has been made in the area of pain management. Cancer pain remains undertreated (Paice & Ferrell, 201). This is due to a multitude of factors: inadequate knowledge of pain physiology and pain management, misconceptions regarding opioids, and unwarranted fear of opioids and addiction, among others.

A relatively recent trend in the clinical management of pain is a focus on prevention (Paice & Ferrell, 2011). Several groups of medications may be used to prevent the pain associated with cancer treatments, namely, nonsteroidal anti-inflammatory drugs (NSAIDs), opioids, and the antiepileptic drugs (AEDs).

Chemotherapy and/or radiation treatment given to eradicate cancer also affect cells that are multiplying rapidly. For patients undergoing treatment for head and neck cancer, a resulting painful mucositis may occur. Attempts at preventing or minimizing this pain with AEDs may result in the use of decreased dosages of opioids.

The purpose of this article is to review the physiology of pain transmission, describe studies on the use of AEDs in pain prevention, and model the application of this strategy to the care of patients with head and neck cancer.

## PAIN TRANSMISSION

In the acute setting, pain protects the body from harm by acting as a warning. Pain occurs as a result of stimulation of a group of receptors called nociceptors. Nociceptors, which are located at nerve endings of C and Aδ fibers, are activated by thermal, mechanical, or chemical stimulation. These noxious stimuli are converted into electrical activity within the peripheral nervous system—a process referred to as transduction. As stimulation of the nociceptor occurs, sodium, potassium, and calcium rush into the area and cause depolarization of the cell. This results in transmission of the impulse along the nerve to the level of the dorsal horn. The rapidity of the transmission depends upon the type of nerve fiber affected (myelinated vs. unmyelinated). The intensity of the impulse depends upon the intensity of the stimulus generated. Activation of the nociceptors results in the release of excitatory neurotransmitters, neuropeptides, and neuromodulators into the synapses between cells.

Glutamate is the major excitatory neurotransmitter in the central nervous system (CNS). Activation of the N-methyl-D-aspartate (NMDA) receptor appears to play a role in increasing CNS excitability (Argoff, 2011). Glutamate-induced plasticity is a key step in increasing synaptic efficacy in the dorsal horn of the spinal cord at the synapse between primary afferent terminals and second-order neurons after injury (Chiechio & Nicoletti, 2012). In addition, release of a host of neuromodulating substances occurs and increases the excitability of the neuron and facilitates transmission of the impulse to the brain.

At the level of the dorsal horn, primary afferents synapse with interneurons prior to traveling to the final pathway in the spinal cord (e.g., spinothalamic tract). The afferents release a number of neurotransmitters, resulting in a massive influx of calcium and sodium to a lesser degree. This influx is a trigger for a number of intracellular events that result in activation or deactivation of other receptors within the cell. In the postsynaptic area, there are calcium channels that are comprised of proteins with multiple subunits. These include α1 pore-forming subunits and the modulatory subunits α2δ, α2β, or α2γ. The α2δ subunit regulates the amount and operation of activation and inactivation of the channel. In C nociceptors, the α2δ subunit is dramatically upregulated after nerve injury and plays a key role in injury-related hypersensitivity and allodynia (Basbaum, Bautista, Scherrer, & Julius, 2009).

Inhibitory neurotransmitters also play a large role in pain transmission. Under normal circumstances (i.e., acute pain), gamma amino butyric acid (GABA) and/or glycine are continuously released to decrease the excitability of the interneuron and modulate pain transmission. Often, as a result of severe and/or chronic pain, microglia are activated and release a number of cytokines that result in response to innocuous stimuli that are resultantly perceived as painful. This is referred to as central sensitization (Argoff, 2011).

Activation of descending pain-suppression pathways (referred to as modulation) has the ability to decrease the likelihood that a stimulus is perceived as painful and/or reduce the perceived intensity of pain. Endogenous opioids are essential in this pathway and are released from a number of locations throughout the CNS, where they can inhibit pain signal transmission (Argoff, 2011). In addition, dopamine and serotonin may have anti- or pronociceptive effects, depending upon the subtype and location of the receptors involved in the transmission. Norepinephrine has antinociceptive effects on transmission by causing the release of inhibitory neurotransmitters such as GABA or glycine (Argoff, 2011).

## RESEARCH IN THE USE OF AEDS FOR PAIN PREVENTION

Several classes of medications are used to treat pain. However, the focus of this article is on relevant literature reporting on the use of some of the newer AEDs in an attempt to prevent pain. Just as we ask patients to premedicate prior to known pain-provoking activities, we may be able to use AEDs to prevent or lessen the pain they experience as a result of cancer or its treatment. This represents a change in the manner in which we approach pain in patients with cancer.

Clark et al., 2012) performed a meta-analysis of patients undergoing a variety of surgical procedures receiving preventive analgesia. Preventive analgesia is defined by the investigators as patients receiving varying doses of a medication, in this case AEDs including gabapentin or pregabalin (Lyrica), during the perioperative period with the intention of preventing or decreasing pain. Dosing and schedules of the medications varied widely in the studies reviewed. Surgical procedures included herniorrhaphy, total joint replacements, cardiac surgery, thoracotomy and breast cancer surgery. Perioperative administration of AEDs resulted in a decrease in chronic postsurgical pain (defined as pain that persisted more than 2 months after the surgical procedure) in 50% of the studies involving gabapentin and 100% of the studies involving pregabalin. In addition, a decrease in postoperative pain scores and opioid consumption was noted in these groups.

Eroglu et al. (2009) demonstrated that gabapentin antagonizes the α2δ1 thrombospondin binding that is necessary for the development of new synapses in the CNS. This is especially true in the setting of injury, when the synapse may undergo remodeling. Reactive astrocytosis is prominent in the spinal cord after peripheral nerve injury leading to pain. Therefore, increased levels of α2δ1 may lead to increased transmission of neuropathic pain impulses. Dolphin (2012) demonstrated that increased α2δ1 levels lead to enhanced excitatory synapse activity by enhancing calcium trafficking and, ultimately, an increase in neuropathic pain. Bauer et al. (2010) echoed the previous authors and also found that pregabalin had effects in previously established synapses, whereas gabapentin did not.

Bar Ad et al. (2010) published a retrospective review of head and neck cancer patients undergoing intensity-modulated radiation therapy (IMRT). Patients received an escalating dose of gabapentin (maximum of 2,700 mg per day or 1,800 mg per day for patients 65 years and older) starting week 2 of treatment and prior to the development of mucositis. Eighty percent of patients treated with IMRT developed at least grade 2 mucositis. Of those who developed mucositis, only 35% required opioids during the last weeks of treatment. The author also pointed out that gabapentin is safe and well tolerated and resulted in few, mild side effects.

## CASE STUDY

Mr. D was a 47-year-old male diagnosed with locally advanced poorly differentiated squamous cell cancer of the oropharynx. The tumor was presumed to be positive for HPV based on P16 testing. P16 is an indirect measure of HPV with a 90% sensitivity (Begum et al., 2003). Positron emission tomography (PET) scan demonstrated a mass 5.9 × 4.2 × 4.6 cm in size centered within the soft palate with extension into the posterior oropharynx and superiorly into the nasopharynx and retropharyngeal/prevertebral space and bilateral longus capitis muscles extending from level of the clivus to C1. Near complete effacement of the oropharynx was present. There was no pathologic lymphadenopathy by imaging criteria; however, evaluation of the lymph nodes was suboptimal due to motion artifact.

On physical examination prior to the start of treatment, Mr. D was in no apparent distress and had a pleasant disposition. Neurologic examination was unremarkable, with pupils equal and reactive to light, extraocular movements intact, and cranial nerves II through XII grossly intact. He was unable to breathe through his nose. He was also unable to taste or smell. Nasopharyngolaryngoscopy was aborted due to complete obstruction of the nasopharynx. Visualization of the posterior oropharynx was also limited due to tumor partially obstructing the posterior oropharynx. No cervical, supraclavicular, or axillary adenopathy was noted. Lungs were clear to auscultation with equal breath sounds. Cardiac exam revealed a regular rate and rhythm, no murmurs or rubs. His abdomen was soft, nontender with positive bowel sounds. The extremities revealed no edema, 5/5 strength and +2 deep tendon reflexes in all extremities, and no spinal tenderness to palpation.

Mr. D was not a surgical candidate. As a result, he received definitive treatment with cisplatin 100 mg/m^2^ every 3 weeks and IMRT to a total dose of 70 Gy to the primary tumor and bilateral neck over 7 weeks. The treatment field for IMRT extended from the base of the skull down to the level of the clavicles with bilateral neck fields. Given the treatment field, Mr. D was anticipated to experience grade 3 mucositis of the nasopharynx and oropharynx.

To prevent pain in Mr. D’s case, gabapentin was initiated at the beginning of week 2 of treatment prior to the development of significant mucositis. By week 4 of treatment, he had grade 3 mucositis of his palate bilaterally as well as his pharyngeal wall, base of tongue, and epiglottis. Mr. D reported intermittent mild pain (maximum rating of 3/10 in the last 2 weeks of treatment) in his throat, described as sharp in quality. This pain description was reported throughout his treatment and recovery period.

Mr. D repeatedly declined opioids but stated that he had no opposition to using them if needed. Although the decision was made not to place a prophylactic feeding tube, Mr. D developed a metallic taste in his mouth that interfered with his ability to eat. As such, he lost more than 10% of his body weight during treatment and required placement of a percutaneous feeding tube (Koyfman & Adelstein, 2012; McLaughlin et al., 2010; Locher et al., 2011). However, he stated that odynophagia (pain with swallowing) did not interfere with his ability to swallow. He experienced grade 2 dysgeusia during treatment. He was also treated for thrush during active CT/RT, with no escalation in his report of pain.

## DISCUSSION

The use of gabapentin to prevent pain was considered successful in Mr. D’s case. Given the size of his radiation field and the dose of radiation received, it is unusual for a patient not to require any opioids during or after treatment. Patients undergoing this treatment frequently experience moderate to severe pain due to oral mucositis, requiring significant doses of opioids to control their pain and maintain oral intake or the ability to maintain some swallowing function. Other measures such as topical lidocaine and rinses containing a mixture of medications that vary among institutions (often referred to as Magic Mouthwash) are also used, yet topical measures are generally inadequate to control pain without the addition of systemic opioids.

In addition, patients are at risk for infection due to bone marrow suppression from chemotherapy. Aspiration pneumonia is also a risk for these patients. This is due to the presence of a large amount of thick saliva caused by irritation of the salivary glands as well as decreased sensitivity of the throat to aspiration, as a result of the radiation. Therefore, NSAIDs are not an ideal pain intervention for this group of patients, as they will mask fevers that would otherwise indicate a potential infection. Gabapentin seems to be an ideal medication for the prevention and treatment of pain due to mucositis caused by treatment with chemotherapy and radiation therapy. Gabapentin is not highly protein-bound or metabolized by the liver; therefore, significant drug interactions are not anticipated. The majority of the medication is excreted unchanged by the kidneys (Pfizer, 2013).

Gabapentin is started at 600 mg at bedtime and escalated over a period of 6 days. Ideally, patients are titrated to a dose of 1,800 to 2,700 mg of gabapentin in divided doses every 8 hours from the start of week 2 of treatment prior to the development of painful mucositis. The dose is escalated slowly over a week to avoid unwanted side effects such as sedation and/or dizziness. Dosing is modified for patients 65 or older due to the potential for greater side effects in elderly patients (Pfizer, 2013). Patients reporting adverse events such as persistent sedation or cognitive impairment may decrease the dose of their medication under the direction of their providers. When decreased, doses should be reduced gradually in order to determine the ideal dose with the fewest side effects.

Patients are monitored for the most common adverse events such as sedation, dizziness (anticipated to be transient in nature), impaired cognition, nausea, swelling of the lower extremities (unrelated to cardiac function), and rash. While chemotherapy is more commonly associated with rash, there is a risk of Stevens-Johnson syndrome with all AEDs; therefore, any generalized rash will result in the discontinuation of gabapentin. Other side effects listed in the package insert are much less common.

Education regarding gabapentin for the patient and family member includes the purpose of the medication, common side effects, dosing schedules, and other interventions that may be utilized if the AED is insufficient to treat the pain experienced by the patient. The need to begin the medication prior to the development of pain is stressed to patients, as it has been demonstrated that gabapentin exerts its effect best prior to the development of new synapses (Clark et al., 2012).

## SUMMARY

Patients undergoing treatment for head and neck cancer experience considerable pain during and following treatment. Gabapentin and pregabalin have a potential role in pain prevention for these patients. These medications have the following actions that may decrease pain due to cancer or its treatment:

- Inhibition of the actions of the A₂ subunit protein (α2δ1)
- Inhibition of glutamate release
- Inhibition of the activity of NMDA receptors
- Inhibition of the activity of voltage-gated sodium channels
- Enhancement of the activity of voltage-gated potassium channels

Inhibitory processes are enhanced, potentially resulting in a decreased incidence of chronic pain, after prolonged exposure (Clark et al., 2012). The actions associated with these medications have the potential to alter the transmission of painful stimuli in patients expected to experience pain as a result of the cancer or its treatment.

## CONCLUSIONS

Head and neck cancer and its treatment frequently result in the development of pain. A large body of literature exists on the best way to treat pain once it occurs. However, literature suggesting that there may be a way to alter how the body transmits painful stimuli in some cases is emerging. This may be accomplished by altering the microenvironment between cells, thereby decreasing the transmission of painful impulses using AEDs. In some cases, this may be best accomplished prior to the development of pain.

The previous discussion outlined the use of gabapentin in head and neck cancer and pregabalin in the surgical population prior to the development of pain as well as the use of the agents in achieving some degree of preventive analgesia. Future research is needed to demonstrate the effectiveness of this class of medications in pain prevention for patients expected to experience pain as a result of cancer and/or its treatment. Studies may include a direct comparison to standard therapy and determination of the appropriate population, as well as ideal dosing and schedules for both gabapentin and pregabalin. 
